# Synchronization and Random Triggering of Lymphatic Vessel Contractions

**DOI:** 10.1371/journal.pcbi.1005231

**Published:** 2016-12-09

**Authors:** James W. Baish, Christian Kunert, Timothy P. Padera, Lance L. Munn

**Affiliations:** 1 Department of Biomedical Engineering, Bucknell University, Lewisburg, Pennsylvania, United States of America; 2 Department of Radiation Oncology, Massachusetts General Hospital and Harvard Medical School, Boston, Massachusetts, United States of America; 3 AMGEN, Cambridge, Massachusetts, United States of America; Imperial College London, UNITED KINGDOM

## Abstract

The lymphatic system is responsible for transporting interstitial fluid back to the bloodstream, but unlike the cardiovascular system, lacks a centralized pump-the heart–to drive flow. Instead, each collecting lymphatic vessel can individually contract and dilate producing unidirectional flow enforced by intraluminal check valves. Due to the large number and spatial distribution of such pumps, high-level coordination would be unwieldy. This leads to the question of how each segment of lymphatic vessel responds to local signals that can contribute to the coordination of pumping on a network basis. Beginning with elementary fluid mechanics and known cellular behaviors, we show that two complementary oscillators emerge from i) mechanical stretch with calcium ion transport and ii) fluid shear stress induced nitric oxide production (NO). Using numerical simulation and linear stability analysis we show that the newly identified shear-NO oscillator shares similarities with the well-known Van der Pol oscillator, but has unique characteristics. Depending on the operating conditions, the shear-NO process may i) be inherently stable, ii) oscillate spontaneously in response to random disturbances or iii) synchronize with weak periodic stimuli. When the complementary shear-driven and stretch-driven oscillators interact, either may dominate, producing a rich family of behaviors similar to those observed *in vivo*.

## Author Summary

For decades, cardiovascular physiology has been an area of intense research, and we have a fundamental understanding of the mechanisms the heart uses to drive blood flow through the distributed network of vessels in the body. The lymphatic system is now receiving similar attention as more is learned about its functional role in disease processes. The importance of the lymphatic system in collecting excess fluid from tissues and returning it to the blood is well known, but how the lymph flow is regulated without a central pump is poorly understood. Each segment of collecting lymphatic vessel can independently contract yielding a network of distributed pump/conduits. This paper shows how the lymphatic muscle cells that squeeze fluid along the lymphatic vessels can be effectively regulated using only chemical and mechanical signals that they receive from their immediate microenvironment. Using stability theory and the tools of nonlinear dynamics we identify two complementary oscillators that respond to stretch of the vessel wall and shear of fluid flowing over the vessel wall. Numerical simulations of the combined oscillators show that they have characteristics well suited to the regulation of distributed systems in general and may have application in other biological and physical contexts.

## Introduction

**Fig 1:**
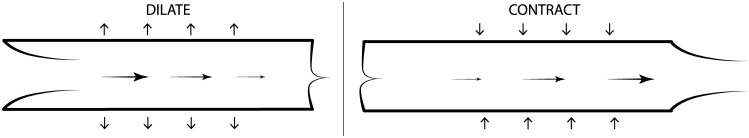
A single lymphangion while opening and closing with valves at the inlet and outlet.

To maintain fluid homeostasis, interstitial fluid drains into the lymphatic system through initial lymphatic vessels that carry it to the collecting lymphatic vessels. The collecting lymphatic vessels transport the fluid (known as lymph) both passively and actively to lymph nodes and back to the systemic blood circulation. Collecting lymphatic vessels are surrounded by specialized lymphatic muscle cells (LMCs) [1] and sub-divided by valve structures that define individual segments called lymphangions (Fig 1) [2]. Lymphangions serve as both pumps and conduits. In contrast to the blood circulation, where a single pump drives flow through relatively passive conduits, each lymphangion has the ability to pump lymph through the converging network to lymph nodes and eventually to the thoracic duct. Pumping occurs when expansions in radius draw fluid into the upstream end of the lymphangion and then expel it downstream during a contraction. Directional flow is enforced by intraluminal valves, which favor flow toward the thoracic duct. Lymphatic vessel contractions are triggered when cytosolic Ca^2+^ entering from intravascular stores and outside the cell surpasses a threshold concentration in the cytoplasm of the LMC, resulting in actin and myosin cross-bridging within the LMCs [3]. The contraction phase ends as transmembrane pumps restore cytoplasmic Ca^2+^ concentration to equilibrium allowing actin-myosin binding to relax, and the trans-wall pressure and the passive elastic properties of the wall to reopen the vessel. The effects of Ca^2+^ on LMC contraction are moderated by endothelial-derived relaxation factors (EDRFs) that act as potent dilators of lymphatic and blood vessels when produced by the vessel-lining lymphatic endothelial cells (LECs) in response to dynamic fluid shear stresses. The best known EDRF is nitric oxide although others such as histamine have been shown to be important [4, 5]. For notational simplicity we represent the entire class of EDRFs herein as NO. The NO and Ca^2+^ levels are both subject to mechanical regulation; Ca^2+^ can enter the cell through stretch-activated ion channels [6, 7], and NO is produced by LECs when they are exposed to increased fluid shear stress [8]. Although rhythmic contractions can be produced by purely chemical oscillations in Ca^2+^ within the LMCs [9, 10], it is likely that feedback regulation is necessary for robust homeostasis. Indeed, we previously used a relatively complex numerical simulation of lymphatic pumping to demonstrate that a wide spectrum of oscillatory behaviors is possible, and that the behavior is very sensitive to local levels of stretch and stress [11].

Our present aim is to reduce the complexity of our previous model to examine how the observed oscillations arise from the integration of simple mechanical and chemical processes within a physiological system. Once simplified, we employ tools such as linear stability analysis to identify key parameter groups that determine the qualitative dynamic behaviors of such systems. Linear stability analysis seeks to determine which parameter values cause a small disturbance to grow rapidly from an initial state, or alternatively decay back to equilibrium. In addition, our approach allows us to show how oscillations can arise from the interactions between mechanical and chemical processes that lack intrinsic oscillators when considered separately. Here we develop generic formulations of the mechano-chemical processes in the muscular lymphatic vessel wall based on Ca^2+^ and NO signaling, then explore the dynamics of each chemical species while holding the effects of the other constant. Finally we examine the behavior of the fully coupled system. Linear stability analysis reveals a new class of oscillator arising from the dynamics of shear and NO that can act alone or in concert with the better recognized Ca^2+^ dynamics. The most remarkable feature of the shear-NO mechanism is its ability to offer distributed control of the pumping process, which is essential for managing a decentralized network of pumps and conduits.

## Methods

### Model Formulation

Our model is based on a single lymphangion (Fig 1) bounded at each end by one-way valves. The radius of the lymphangion is governed by the radial forces which are determined by the contractile (Ca^2+^) and dilatory (NO) signaling molecules. Neglecting inertial effects, the radial forces on the vessel wall balance as

$$D\frac{dR}{dt}=-E(R)-F(R,C_{Ca},C_{NO})+A_{R}(t)$$ (1)

where the left hand side represents rate dependent effects with *D* incorporating the visco-elastic material properties of the vessel wall as well as viscous losses in the flow and lags in the transduction of concentrations into force, E is a restoring force-including elastic forces and vessel “tone” -imparted by the material properties of the vessel wall and F is a dynamic inward acting contractile force produced by muscle cells that surround the vessel. To a first approximation, we assume that the concentration of Ca^2+^ is transduced into a contractile force as $F=F_{Ca}C_{Ca}/(1+\alpha C_{NO})$ where *α* scales the possible desensitizing effect of NO [12]. The activation term $A_{R}$ can include a steady component from the mean transmural pressure difference *p*_*m*_ that influences the baseline radius as well as extrinsic disturbances to the radius from the surrounding tissue and adjacent lymphangions.

The restoring force is typically highly nonlinear[13–15]. Here we adopt the form $E(R)=Ae^{aR}-p_{offset}$ with stiffening coefficient *a*, scaling coefficient *A*, and offset pressure *p*_*offset*_ selected to give a good fit of Shirasawa and Benoit (see the third figure of reference [15]) at typical operating pressures. In our numerical simulations we retain the full nonlinear form of E(R), but for the stability analysis that follows we linearize the elastic force near an equilibrium radius *R*_1_ in the absence of dynamic increments to Ca or NO as $E(R)\approx E_{0}+E_{1}(R-R_{1})$ where *E*_0_ is the elastic force at equilibrium and a Taylor series expansion near equilibrium yields $E_{1}=Aae^{aR_{1}}$. We find the equilibrium radius by solving $0=D\frac{dR}{dt}=-(Ae^{aR_{1}}-p_{offset})-F_{Ca}S_{Ca_{0}}/K_{Ca}+p_{m}$ for *R*_1_ where *p*_*m*_ is the mean transmural pressure. Given the stiffening behavior of the wall ($E_{1}\propto e^{aR_{1}}$), we expect that appropriate values of *E*_1_ will be larger at higher mean transmural pressures where the equilibrium radius will be somewhat larger.

The concentrations of the signaling molecules (*i* ∈ {*Ca*, *NO*}) are governed by the generic conservation law

$$\frac{dC_{i}}{dt}=-K_{i}(C_{Ca},C_{NO})+S_{i}(R,\dot{R},C_{Ca},C_{NO})+A_{i}(t)$$ (2)

where all concentrations are taken to be dimensionless ratios relative to a suitable reference, $K_{i}$ is clearance of the signaling species through chemical reaction, transmembrane ion pumps and advective-diffusive transport, $S_{i}$ is a dynamic source term for the signaling molecule and $A_{i}$ is an additional source term that can include the effects of imposed flow from upstream fluid pressure, inflammation, pace-making signals from adjacent cells, neural signaling, random disturbances, etc.

### Stretch-Ca Dynamics

Since our focus is on the interactions between Ca^2+^ and NO, we introduce a minimal representation of the Ca^2+^ dynamics rather than a fully detailed model of Ca^2+^ oscillations as may be found in the literature [10, 16]. We retain the following features: i) at rest, Ca^2+^ is at a low concentration in the cytoplasm of LMCs; ii) a contraction is initiated when Ca^2+^ is rapidly admitted to the cytoplasm through ion-selective channels thereby triggering cross bridge formation between actin and myosin chains creating a contractile force [17]; iii) relaxation of LMCs coincides with a drop in cytoplasmic Ca^2+^ concentration due to a drop in the rate of influx and the restoration of baseline conditions by ion pumps in the cell membrane and sarcoplasmic reticulum; and iv) the LMC is refractory to a new contraction cycle until Ca^2+^ levels have returned to near equilibrium. As the Ca^2+^ levels approach their threshold level, we hypothesize that the membrane acquires sensitivity to small perturbations. Furthermore, the sensitivity is enhanced when the membrane is stretched to a larger radius. This models stretch-sensitive ion channels found in LMCs [6, 18]. Each step in the process has the potential for modulation by NO. Alternatively, each form of modulation by NO can be disabled to demonstrate behaviors that have been observed in experimental preparations, for example after removal of LECs (which produce NO) or the genetic or pharmacological suppressions of NO [19–21].

We mathematically express the release of Ca^2+^ into the cytoplasm from intracellular stores and the extracellular fluid as the sum of a steady source *S*_*ca*0_ needed to maintain the baseline Ca^2+^ concentration and a transient component that is sufficiently rapid to be modeled as an impulse function *δ*(*t*) where *t* is the time since the Ca^2+^ concentration most recently passed the threshold necessary to trigger another contraction *C*_CaThresh_. This is expressed as,

$$S_{Ca}=S_{Ca0}+\frac{S_{Ca1}\delta (t)}{\left(1+\gamma C_{NO}\right)}$$ (3)

where *S*_*Ca*1_/(1 + *γC*_*NO*_) is the magnitude of a bolus of Ca^2+^ with a possible reduction due to NO that is scaled by *γ*. We model the clearance of Ca^2+^ from the cytoplasm with

$$K_{Ca}=K_{Ca}(1+\beta C_{NO})(C_{Ca}-C_{\text{CaThresh}})$$ (4)

where *K*_*Ca*_ is a rate constant and *β* scales the possible enhancement of Ca^2+^ clearance attributed to NO [12, 16]. At high concentrations of Ca^2+^ the clearance rate may be limited by the membrane pump capacity, but near the threshold required to trigger a contraction we assume clearance rates proportional to the concentration increment. The threshold itself may include a random component that we incorporate into the activation term.

The linearized form of the model for a constant level of NO can now be written as

$$D\frac{dR}{dt}=-E_{1}R-F_{Ca}C_{Ca}+A_{R}(t)$$ (5)

and

$$\frac{dC_{Ca}}{dt}=-K_{Ca}C_{Ca}+S_{Ca1}\delta (t)+A_{Ca}(t)$$ (6)

where the constants have been absorbed into the activation terms so that the radius and concentration now represent the increments from baseline values. The parameters *S*_*Ca*1_ and *K*_*Ca*_ now include the adjustments due to NO introduced in Eqs 3 and 4.

### Shear-NO Dynamics

In the previous section we developed the model so that it reproduces Ca^2+^ induced contractions. We next considered how NO, created when LECs experience increased shear stress, can modulate contractions when it diffuses rapidly into adjacent LMCs. A suitable form for the NO source term can be obtained by considering steady laminar flow in a circular tube with negligible inertia [22]. Conservation of mass in a tube of time-varying radius requires that $\frac{\partial Q}{\partial z}=-2\pi R\frac{dR}{dt}$ which when integrated with respect to *z* along a vessel yields $Q(z)=-2\pi R\frac{dR}{dt}z+C_{1}$ where the constant of integration depends on the end conditions for the lymphangion. When the segment is contracting $\frac{dR}{dt}<0$ and the upstream valve is closed [*Q*(0) = 0] we have $Q(z)=-2\pi R\frac{dR}{dt}z$. Alternatively, if the segment is expanding $\frac{dR}{dt}>0$ and the downstream valve is closed (*Q*(*L*) = 0), we obtain $Q(z)=2\pi R\frac{dR}{dt}(L-z)$. We can express the mean flow along the length more compactly as $\bar{Q}=\pi R\left|\frac{dR}{dt}\right|L$. When additional flow *Q*_0_ is imposed on the segment by an axial pressure gradient we have $\bar{Q}=\pi R\left|\frac{dR}{dt}\right|L+Q_{0}$. Approximating the velocity profile with that of steady laminar flow with negligible inertia [22] we relate the mean shear stress *τ* to the flow rate by $\tau =\frac{4\mu \bar{Q}}{\pi R^{3}}$. The mean shear stress along the segment due to dynamic changes during contraction or expansion is therefore approximately $\tau =\frac{4\mu L}{R^{2}}\left|\frac{dR}{dt}\right|+\frac{4\mu Q_{0}}{\pi R^{3}}$. Recent studies show that valves in collecting lymphatic vessels are biased toward the open condition [23], but the simplification employed here allows us to study the basic stability of the system, at the possible expense of some accuracy in the predictions of pumping efficiency. There may be levels of shear stress below which NO production is negligible and above which NO production saturates at a maximum, but here we linearize the transduction of shear stress into the production of NO in an intermediate range to yield

$$S_{NO}=\frac{S_{NO}}{R^{2}}\left|\frac{dR}{dt}\right|+S_{NO_{0}}$$ (7)

where *S*_*NO*_ has absorbed the remaining constants in the shear stress expression and $S_{NO_{0}}$ represents NO released due to the through-flow term *Q*_0_ or chronic sources of NO such as might arise during inflammation. The source term $S_{NO_{0}}$ can be time varying, but arises from the local environment of the lymphangion and mathematically acts as an input to our model of a single lymphangion rather than as an interaction within the system itself. $S_{NO_{0}}$ therefore can serve as an external trigger to the system or as a steady offset, but does not directly impact the dynamics of an individual lymphangion, except by parametrically (rather than dynamically) changing the equilibrium radius.

We can examine the effects of fluid viscosity on the pressure by using the same set of assumptions. The pressure will vary due to viscous flow effects according to $\frac{\partial p}{\partial z}=\frac{-8\mu Q}{\pi R^{4}}$. When contracting we have $\frac{\partial p}{\partial z}=\frac{16\mu }{R^{3}}\frac{dR}{dt}z$, which when integrated along the length gives $p(z)=\frac{16\mu }{R^{3}}\frac{dR}{dt}z^{2}+p(0)$. Averaging over the length of the segment yields $\bar{p}=\frac{16\mu L^{2}}{3R^{3}}\frac{dR}{dt}+p(0)$ where the first term gives the magnitude of the pressure decrement (or increment for vessel expansion) due to flow induced by the contraction of a single lymphangion. We see that the pressure increment due to flow induced by the single lymphangion also multiplies $\frac{dR}{dt}$, so it can be absorbed into the overall damping term *D*. For typical vessel sizes, we find that the lag due to viscosity is orders of magnitude smaller than that from chemical and mechanical lags which are on the order of one second.

NO does not produce a true outward force. However, it is conceptually equivalent to consider an effective force produced by NO that has the effect of countering $F_{Ca}$ and the elastic effects. Mathematically, for small *αC*_*NO*_, we can write this as

$$F=\frac{F_{Ca}C_{Ca0}}{1+\alpha C_{NO}}\approx F_{Ca}C_{Ca0}(1-\alpha C_{NO})$$ (8)

By defining *F*_*NO*_ ≡ *F*_*Ca*_*C*_*Ca*0_*α*, we can write

$$F_{NO}=-F_{NO}C_{NO}$$ (9)

And the net force from Eq 8 becomes

$$F=F_{Ca}C_{Ca0}-F_{NO}C_{NO}$$ (10)

where the net contractile force is decomposed into a positive term set by the baseline Ca^2+^ levels and a negative term that represents how the Ca^2+^ levels are modulated by NO. Thus, the NO-dependent term is not a true outward force, but arises mathematically from a reduction in the Ca^2+^-dependent contractile forces.

The parameter values used in the simulations that follow are given in Table 1. The parameter values were based on experimental data where possible, but were chosen to demonstrate a wide range of mathematical behaviors for the system rather than to mimic a particular experimental data set in detail. As a representative example, we show simulations based on measurements in rats [24] which offer data relating Ca^2+^ concentrations to lymph vessel diameter and contractile tension. Our own experiments discussed later [20] were done on mice which have smaller collecting lymphatic vessels than rats.

**Table 1:** Baseline Parameter values

Parameter	Symbol	Value	Units	Source
Lymphangion radius	*R*_0_	1.3x10^-4^	m	[15]
Mean pressure	*p*_*m*_	100–1000	Pa	[15]
Wall stiffening exponent	*a*	2.45x10^4^	m^-1^	[15]
Wall stiffening coefficient	*A*	12.3	Pa	[15]
Offset pressure for wall stiffness	*p*_*offset*_	100	Pa	[15]
Wall damping coefficient	*D*	3x10^6^	Pa-s/m	[15]
Baseline Ca concentration	*C*_*Ca*0_	1		
Baseline Ca release rate	*S*_*Ca*0_	1		
Ca release pulse size	*S*_*Ca*1_	0.85		[15]
Ca force production coefficient	*F*_*Ca*_	100	Pa	[15]
NO source from shear stress coefficient	*S*_*NO*_	3x10^-3^	m	
Ca clearance rate constant	*K*_*Ca*_	1	s^-1^	[15]
NO clearance rate constant	*K*_*NO*_	5	s^-1^	
Contraction force suppression by NO	*α*	0–1		
Ca clearance enhancement by NO	*β*	0–1		
Ca source blunting by NO	*γ*	0–1		
Ca noise standard deviation	*σ*_*Ca*_	10^−3^		
NO noise standard deviation	*σ*_*NO*_	10^−3^		
Integration time step	dt	10^−4^	s	

Parameters without units are taken to be dimensionless ratios. The concentrations are normalized relative to nominal concentrations. Force related terms are given as pressure equivalents in a circular tube.

Specific parameters governing the effects of NO are difficult to estimate, but fortunately may not be necessary here. As will be shown in the results section, we require only estimates of combinations of parameters such as *S*_*NO*_ and *F*_*Ca*_*C*_*Ca*0_*α*, rather than values for each parameter individually. Unlike the geometrically-detailed continuum model of lymphatic NO transport in Wilson et al [25] that includes shear-induced production and clearance by diffusion, convection and reaction, our present model employs averages over a single lymphangion and combines the sensitivity to shear stress with the rate of production of NO. To that end, we employ parameter values that yield diameter changes due to NO on the order of 10% as observed in [20, 21]. Moreover, we expect the effects of NO to be rapid. NO is released by endothelial cells about 2 seconds or less after increases in shear stress as observed previously [26–29]. Lymphatic vessels can be expected to dilate faster than blood vessels [30] because their muscle cells contain more rapid-acting contractile proteins than those of blood vessels [31]. We take the clearance of NO to be similar to, but somewhat faster than, that of Ca^2+^ [32]. Parameters such as the contractility, NO production and the mechanical stiffness appear to depend on anatomical location, species and age [13, 28, 33–36], suggesting that the full range of possibilities realizable *in vivo* awaits further investigation.

## Results

Here we first present analytical and numerical results for cases in which the stretch-Ca^2+^ dynamics are active, but with constant NO levels. We will then present results of our model for when shear-NO dynamics are active, but Ca^2+^ levels do not spike, but remain near baseline. Finally, we will present results of our model for fully coupled stretch-Ca^2+^ and shear-NO processes.

### Stretch-Ca^2+^ Dynamics with Constant NO Levels

In the absence of dynamic activation, Eq 6 implies the Ca^2+^ concentration during each contraction cycle will decay as $C_{Ca}(t)=S_{Ca1}e^{-K_{Ca}t}$. Using this as an input to Eq 5, we find that the radius varies from its baseline value during each contraction cycle as

$$\Delta R(t)=\frac{F_{Ca}S_{Ca1}}{E_{1}K_{Ca}}\frac{\left(e^{-t/t_{Ca}}-e^{-t/t_{mech}}\right)}{\left(t_{mech}-t_{Ca}\right)}$$ (11)

where we see that the return to equilibrium depends on two characteristic times, one set by the rate of Ca^2+^ clearance *t*_*Ca*_ = 1/*K*_*Ca*_ and the other by the mechanical lag *t*_*mech*_ = *D*/*E*_1_ which can include the lag between the concentration increase and force production. As a reference time scale we have selected *t*_*Ca*_ = 1/*K*_*Ca*_ = 1 s which is in the range observed by Shirasawa and Benoit [15]. They observed a similar lag between the rise in Ca^2+^ concentration and the peak force generation. Here we use this lag as an estimate of *t*_*mech*_ which we take to incorporate the visco-elastic and chemical-mechanical transduction lags.

While both time constants contribute to the overall response, the slower of the two characteristic times gives the dominant time constant *t*_*c*_ that determines the return to equilibrium. Experimental observations of the magnitude of radius change as a function of pressure show that it decreases with increased internal pressure [21]. This phenomenon is reproduced by our model as the vessel wall stiffens (larger *E*_1_ in the denominator) at greater radius. The amplitude may be further modified if the myosin cross bridging is length dependent as seen in skeletal muscle [36].

The frequency of contractions at constant NO levels is set by the interplay between the characteristic time for calcium *t*_*Ca*_ and the magnitude of the random activation term. In addition to the steady source of Ca^2+^ that establishes the vascular tone, we include a random component $A_{CaRand}(R,t)$ with zero mean and a standard deviation *σ* that can be applied to either the concentration itself or to the threshold level at which a new release of Ca^2+^ is triggered. A higher radius leads to more stretch in the LMC membrane and therefore greater sensitivity of ion channels. This can be modeled by increasing the noise level (for example let *σ* ∝ *p*_*m*_). This will lead to a higher frequency at larger radii as found experimentally [21]. Exponential decay of Ca^2+^ near equilibrium leads to a latency period between contractions that varies as *T* = *t*_*c*_ log(*C*_*max*_/*σ*) where *C*_*max*_ is the magnitude of the Ca^2+^ increment from baseline.

**Fig 2:**
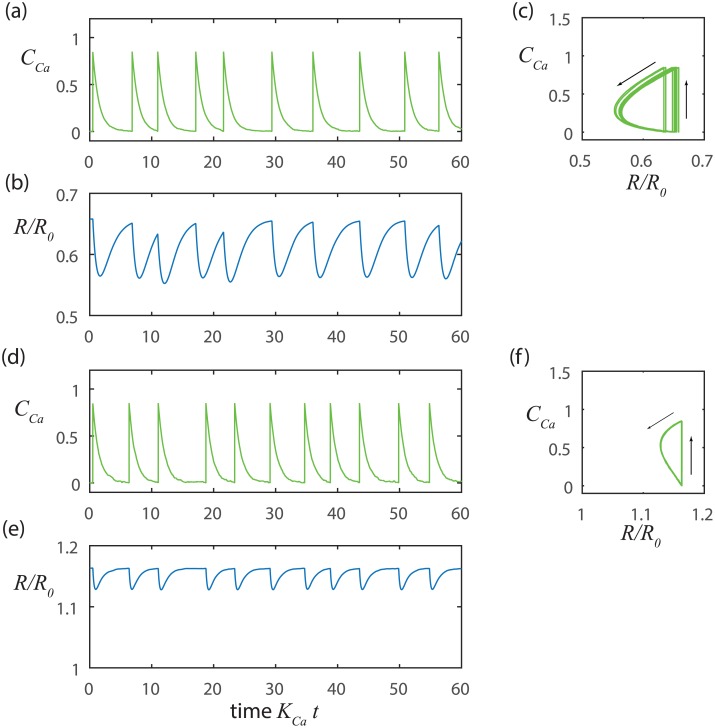
Stretch-Ca dynamics at constant NO with noise triggering. At lower pressure a-c) *p*_*m*_ = 100 Pa, *t*_*Ca*_ = 1s and *t*_*mech*_ = 1.22 s, and at a higher pressure d-f)*p*_*m*_ = 500Pa, *t*_*Ca*_ = 1s, and *t*_*mech*_ = 0.24 s. The noise level is *σ* = 0.01. In each case the overall time constant is given approximately by the greater of *t*_*Ca*_ and *t*_*mech*_. The period is predicted approximately by *t*_*c*_ log(*C*_*max*_/*σ*) where *C*_*max*_ is the amplitude of the change in Ca^2+^ concentration.

We further investigated random activation with the aid of numerical simulations implemented with the Euler-Maruyama method [37], which properly scales the computational time step with the standard deviation of the noise (Fig 2). The simulation presented in Fig 2d–2f results from a higher mean pressure than Fig 2a–2c. Thus, the baseline radius is larger in Fig 2d–2f, which in turn yields a stiffer wall (larger *E*_1_) leading to a smaller mechanical time constant (smaller *t*_*mech*_) and reduced amplitude for change in the radius.

We note that our simple model of stretch-Ca^2+^ dynamics replicates important features of the contraction cycles observed *in vivo* [20] where contractions are generally similar to one another in magnitude and duration, but may be separated by inconsistent periods of latency. In Fig 2, we see that the simulated contractions are nearly identical to each other (that is, the trajectories nearly retrace one another in the phase portraits shown Fig 2c and 2f), but occur on inconsistent intervals. Even though the intervals between contractions are not perfectly uniform, they are well estimated by *t*_*c*_ log(*C*_*max*_/*σ*). We also see that increased transmural pressure can reduce the interval between contractions by stiffening the wall (*p*_*m*_ ↑⇒ *R*_1_ ↑⇒ *E*_1_ ↑⇒ *t*_*mech*_ ↓⇒ *t*_*c*_ ↓⇒ *T*↓) and also by increasing the sensitivity of the Ca^2+^ channels by stretching the vessel wall (*p*_*m*_ ↑⇒ *σ* ↑⇒ *T* ↓) yielding higher frequency contractions (compare Fig 2b and 2e).

**Fig 3:**
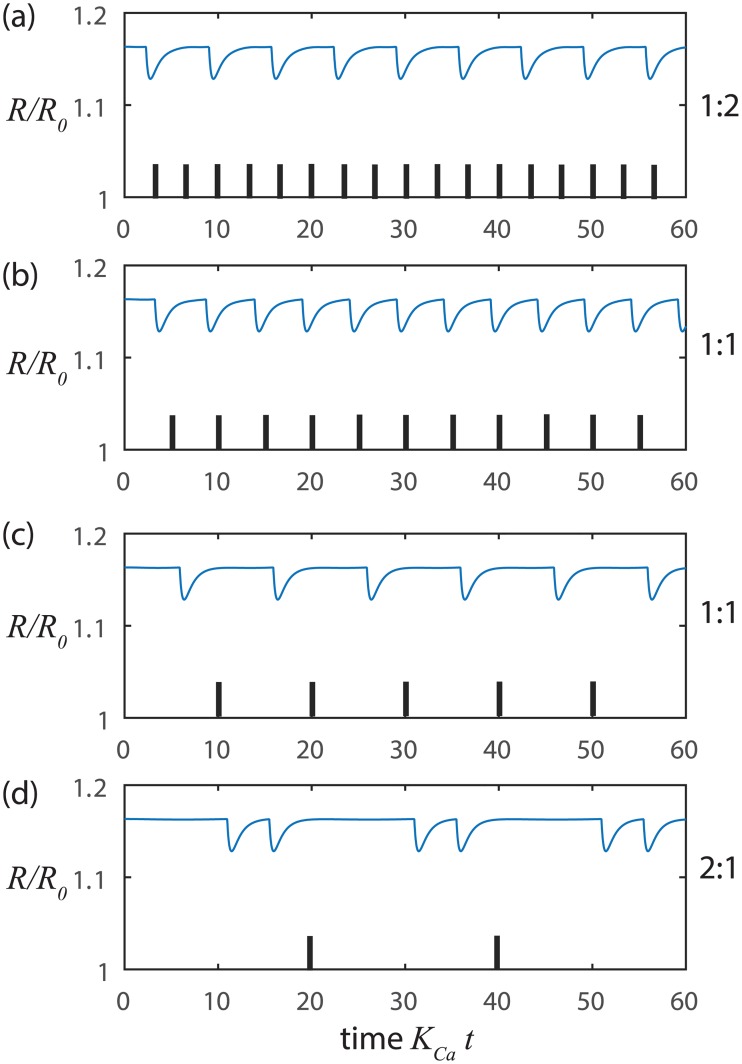
Synchronization of stretch-Ca dynamics with small amplitude sinusoidal inputs at constant NO. At higher pressure *p*_*m*_ = 500 Pa, *t*_*Ca*_ = 1 s, and *t*_*mech*_ = 0.24 s with input amplitude of 0.01 and input frequencies of a) 0.3 Hz, b) 0.2 Hz, c) 0.1 Hz and d) 0.05 Hz. The tick marks indicate the beginnings of successive cycles of the sinusoidal input signal. The ratio of output frequency to input frequency is at the right of each panel.

We also find that our model of the stretch-Ca^2+^ process readily synchronizes when we impose extrinsic rhythmic pace-making since only small variations in the Ca^2+^ concentration relative to the threshold level are needed to initiate the next contraction cycle (Fig 3). Such small variations in Ca^2+^ concentration can be readily introduced by diffusion or voltage signals from adjacent LMCs. Alternatively, the vessel may be locally stretched by lymph arriving from upstream, which can also trigger a local contraction. In this way, neighboring LMCs can synchronize contractions to coordinate flow along a series of lymphangions throughout a connected network of collecting lymphatic vessels [38, 39].

### Shear-NO Dynamics with Ca^2+^ Near Baseline Levels

The conditions for oscillations in radius to arise near baseline Ca^2+^ levels in the absence of sharp spikes in Ca^2+^ as considered in the previous section are available from linear stability analysis of the shear-NO process near a point *R*_1_ which yields

$$\left[\begin{array}{c} \dot{R}\\ \dot{C_{NO}} \end{array}\right]=\left[\begin{array}{cc} -\frac{E_{1}}{D} & \frac{F_{NO}}{D}\\ -\frac{S_{NO}E_{1}}{DR_{1}^{2}} & \;sgn(\dot{R})\frac{S_{NO}F_{NO}}{DR_{1}^{2}}-K_{NO} \end{array}\right]\left[\begin{array}{c} R\\ C_{NO} \end{array}\right]+[inputs]$$ (12)

where the inputs include all extrinsic disturbances from the adjacent lymphangions and surrounding tissue. We treat small variations in *R* parametrically so that the dynamics of the system may be characterized by the eigenvalues of the Jacobian matrix [40] which are roots of the characteristic polynomial:

$$\lambda^{2}+\left(\frac{E_{1}}{D}+K_{NO}-sgn(\dot{R})\frac{S_{NO}F_{NO}}{DR_{1}^{2}}\right)\lambda +\frac{E_{1}K_{NO}}{D}=0$$ (13)

**Fig 4:**
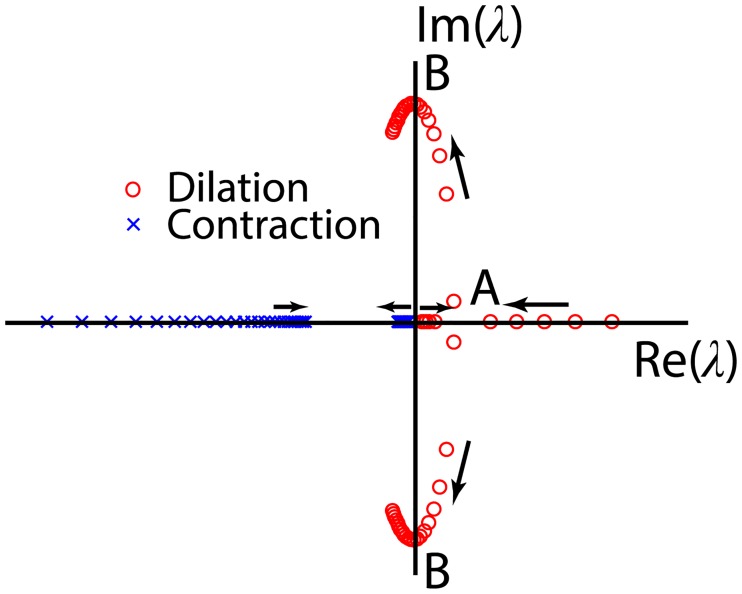
Location of the shear-NO eigenvalues in the complex plane as the baseline radius *R*_1_ increases parametrically. The arrows indicate the direction of increasing *R*_1_. The eigenvalues during contraction always have a negative real part indicating stability. When the baseline radius is large enough to move the eigenvalues beyond point B the system is inherently stable during dilation. At point B the system is marginally stable and will oscillate at frequency *f* = (*E*_1_*K*_*NO*_/*D*)^1/2^/2*π*. For smaller baseline radius, between points A and B, the response is unstable, but oscillatory. And when the baseline radius is smaller at point A, the dynamic component of the radius increases exponentially without oscillation until the radius is large enough to reach the range between A and B where oscillations can occur.

Since all of the coefficients are positive, stability requires only that the second term be positive. Thus the system is always stable during contraction ($\dot{R}<0$). However during dilation ($\dot{R}>0$) the second term can be positive or negative which allows the system to switch between stability and instability (Fig 4).

**Fig 5:**
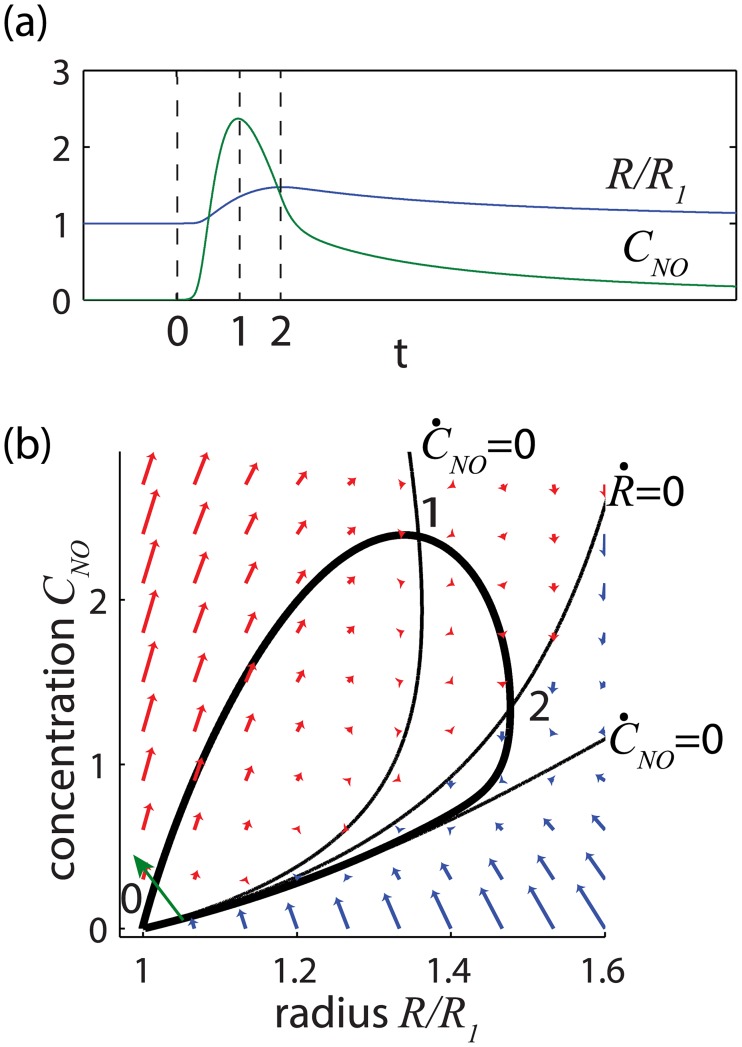
a) Generic time response and b) phase portrait of the shear-NO oscillator. Following a small perturbation shown as a green arrow near equilibrium at point 0, by either a decrease in radius or an increase in concentration, the radius increases unstably (red arrows $\dot{R}>0$) until $\dot{R}=0$ at point 2. Thereafter, the trajectory begins a stable return (blue arrows $\dot{R}<0$) to equilibrium at point 0. NO reaches its peak concentration at point 1 before the radius reaches its maximum, but this point does not directly influence the stability of the system.

Herein is the key feature from which NO can induce spontaneous oscillations in the radius without the sharp spikes in Ca^2+^ concentration described in Eq 6. If the radius is large enough so that *E*_1_/*D* + *K*_*NO*_ > *S*_*NO*_*F*_*NO*_/*DR*^2^, then the fixed point is inherently stable. If instead, the radius is small enough that *E*_1_/*D* + *K*_*NO*_ < *S*_*NO*_*F*_*NO*_/*DR*^2^ then the radius will unstably increase when perturbed. The instability arises because a slight increase in radius (from point 0 on Fig 5) pulls fluid into the lymphangion, increasing shear and temporarily creating a runaway effect wherein more NO is released from the LEC further increasing the radius and drawing in still more fluid (upper branch from point 0 to point 2 on Fig 5b). The instability persists until the radius becomes large enough at point 2 that the shear stresses begin to drop because more cross-sectional area is available for lymph flow. Thereafter the release of NO occurs more slowly than its degradation so that the system can return stably to equilibrium along the lower branch of the trajectory from point 2 to 0. Mathematically, the unstable increase in radius persists until the sign of $\dot{R}$ changes at point 2. A change in the sign of $\dot{R}$ does not require that the eigenvalues move to the left half of the complex plane at point B on Fig 4 as required for inherent stability, but rather requires only that the radius increase sufficiently to move the eigenvalues off of the real axis beyond point A, thus permitting at least a partial cycle of oscillation that includes a time at which $\dot{R}=0$. As the vessel begins to contract, the sign of *S*_*NO*_*F*_*NO*_/*DR*^2^ changes at the R-nullcline where $\dot{R}=0$, leading to an unconditionally stable return to the original radius. The time scale for contraction is approximated by *t*_*c*_ ≈ *t*_*mech*_ + *t*_*NO*_ + *t*_*FNO*_ where the three contributions arise from mechanical lag *t*_*mech*_ as before, the clearance of NO *t*_*NO*_ = 1/*K*_*NO*_, and the rate of force modulation by NO *t*_*FNO*_ = *S*_*NO*_*F*_*NO*_/*E*_1_*K*_*NO*_*R*^2^. To a similar degree of approximation, the instability of the NO-shear dynamics requires *t*_*mech*_ + *t*_*NO*_ ≤ *t*_*FNO*_. In other words, the change in force elicited by shear stress must persist longer than processes that tend to dissipate its effects. Exact algebraic expressions for the eigenvalues may be employed if desired, but this approximation captures the key dependencies. See Table 2.

**Table 2:** Representative Time Constants Based on Parameters in Table 1.

	Equilibrium Radius	Linearized Stiffness	Mechanical Time Constant	Calcium Clearance Time Constant	NO Clearance Time Constant	NO-Shear Time Constant	Overall Time Constant
	*R*_1_(m)	*E*_1_(Pa/m)	*t*_*mech*_(s)	*t*_*Ca*_(s)	*t*_*NO*_(s)	*t*_*FNO*_(s)	*t*_*c*_(s)
*p*_*m*_ = 100 Pa *α* = 1 *β* = *γ* = 0	8.5x10^-5^	2.45x10^6^	1.22	1	0.2	3.35	4.77
*p*_*m*_ = 500 Pa *α* = 1 *β* = *γ* = 0	1.51x10^-4^	1.23x10^7^	0.24	1	0.2	0.21	0.66

**Fig 6:**
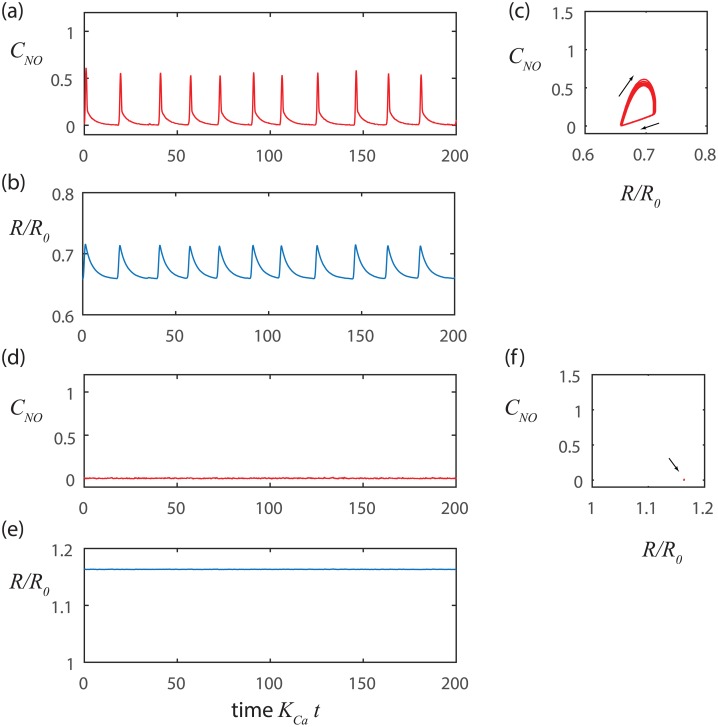
Shear-NO dynamics at constant Ca^2+^ with noise triggering. At low pressure a-c) *p*_*m*_ = 100 Pa, *t*_*Ca*_ = 1 s, *t*_*mech*_ = 1.22 s, *t*_*NO*_ = 0.2 s and *t*_*FNO*_ = 3.35 s where the shear-NO oscillator is unstable (*t*_*mech*_ + *t*_*NO*_ < *t*_*FNO*_) and at a higher pressure d-f) *p*_*m*_ = 500 Pa, *t*_*Ca*_ = 1 s, *t*_*mech*_ = 0.24 s, *t*_*NO*_ = 0.2 s and *t*_*FNO*_ = 0.21 s where the shear-NO oscillator is stable (*t*_*mech*_ + *t*_*NO*_ > *t*_*FNO*_) yielding little change in radius. The noise level is *σ* = 0.01. In each case the overall time constant for return to equilibrium is given approximately by *t*_*mech*_ + *t*_*NO*_ + *t*_*FNO*_. The period is predicted approximately by *t*_*c*_ log(*C*_*max*_/*σ*) where *C*_*max*_ is the amplitude of the changes in NO concentration.

The NO cycle can be generalized into a controllable and synchronizable oscillator. Fig 6 shows the behavior of the NO cycle in response to small random disturbances. During the stable contraction process, the vessel remains refractory to disturbances until close enough to equilibrium for a random disturbance to trigger another cycle, much as we found with the stretch-Ca process. Here the period of the NO-induced oscillations varies as *t*_*c*_ log(*C*_*max*_/*σ*) as before but *t*_*c*_ and *C*_*max*_ now refer to NO rather than Ca^2+^. As with Ca^2+^, a relatively quiet environment or reduced sensitivity to disturbance will elicit longer latency periods between cycles but will not significantly change the shape of the shear-NO cycle.

**Fig 7:**
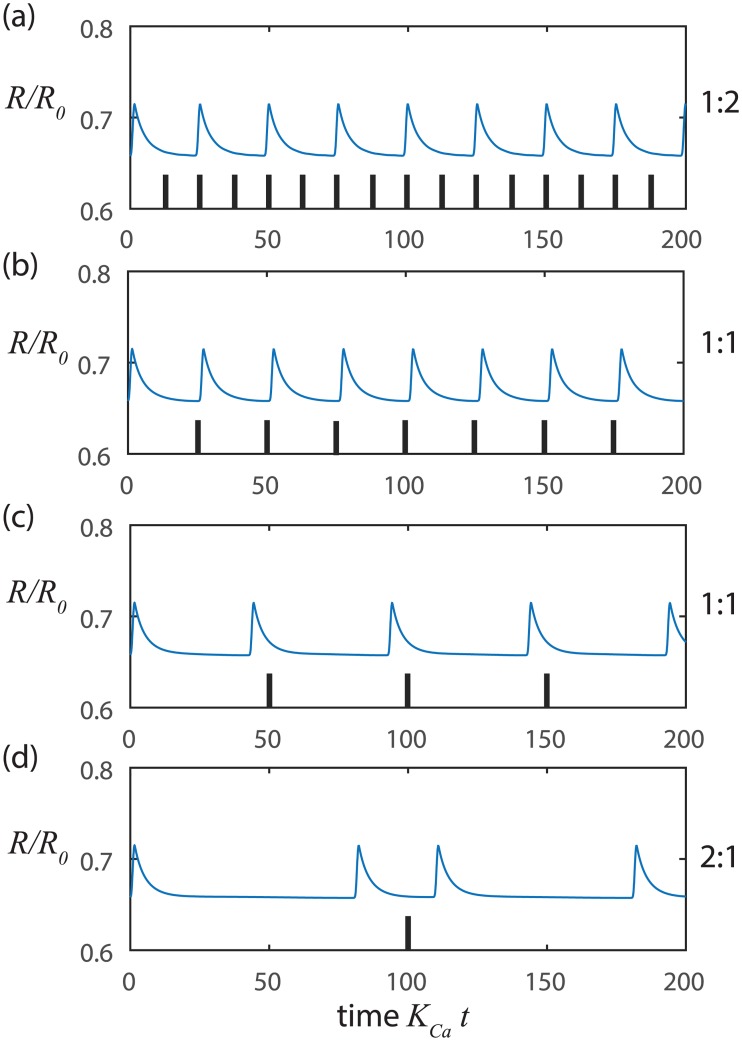
Synchronization of shear-NO dynamics with small amplitude sinusoidal inputs at constant Ca. At lower pressure *p*_*m*_ = 100 Pa, *t*_*Ca*_ = 1 s, *t*_*mech*_ = 1.22 s, *t*_*NO*_ = 0.2 s and *t*_*FNO*_ = 3.35 s where the shear-NO oscillator is unstable (*t*_*mech*_ + *t*_*NO*_ < *t*_*FNO*_) with input amplitude of 0.01 and input frequencies of a) 0.08 Hz, b) 0.04 Hz, c) 0.02 Hz and d) 0.01 Hz. The tick marks indicate the beginnings of successive cycles of the sinusoidal input signal. The ratio of the output frequency to the input frequency is at the right of each panel.

**Fig 8:**
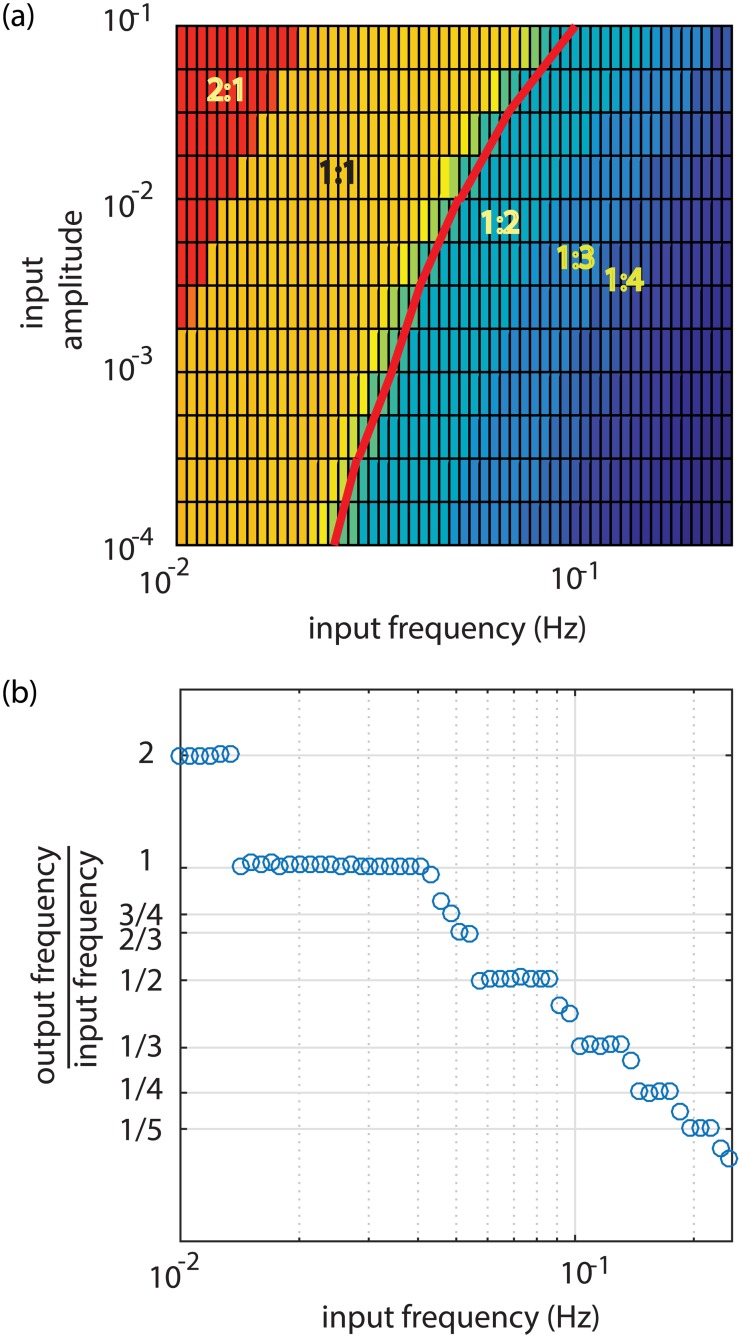
Synchronization of the shear-NO oscillator with a sinusoidal input. a) Color bands indicate domains in which the output and input frequencies lock into simple, integer ratios. The red curve gives an estimate of the autonomous frequency from *f* = 1/(*t*_*c*_*log*(*C*_*NOmax*_/*σ*) where the amplitude of the noise has been replaced by the amplitude of the input sinusoid, b) Output frequencies at input amplitude of 0.01 showing a so-called Devil's staircase of discrete, rational values.

The NO dynamics can also readily synchronize with externally-imposed, small-amplitude sinusoids (Figs 7 and 8). Fig 7b and 7c show how the radius oscillates at precisely the input frequency for frequencies reasonably close to the response when noise triggered (Fig 6). However, when the input frequency is too high (Fig 7a) or too low (Fig 7d) synchronization occurs, but at half or double the input frequency, respectively. Fig 8 shows a parametric study of synchronization over a wide range of input frequencies and amplitudes where we find that synchronization can include a variety of integer ratios between input and output frequencies as explained further in the Discussion.

### Combined Stretch-Ca^2+^ and Shear-NO Dynamics

Having explored the system dynamics when Ca^2+^ and NO are taken to be constant relative to each other we now consider their combined, dynamic effects. Our model includes three possible interactions reported in the literature: NO may i) desensitize the LMCs to Ca^2+^ as modeled by Eq 10, ii) modify the availability of Ca^2+^ by 1/(1 + *γC*_*NO*_) or iii) speed clearance of Ca^2+^ by 1 + *βC*_*NO*_ [41, 42].

**Fig 9:**
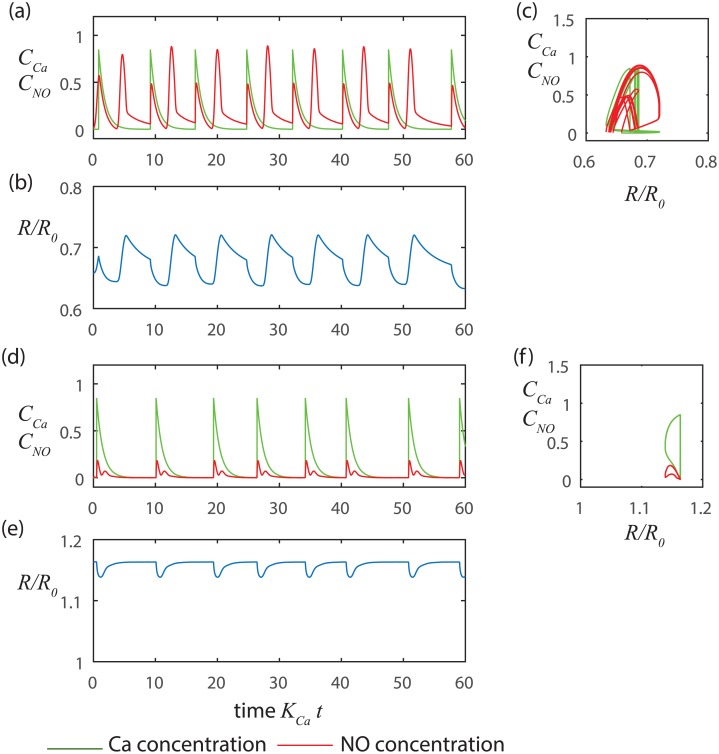
Fully coupled Ca^2+^ and NO dynamics operating autonomously. a-c) Low pressure (small radius) with overshoot of the nominal radius due to instability in the NO dynamics leading to Fig 8 trajectories in phase space (*t*_*mech*_ + *t*_*NO*_ < *t*_*FNO*_) *p*_*m*_ = 100 Pa, *t*_*Ca*_ = 1 s, *t*_*mech*_ = 1.22 s, *t*_*NO*_ = 0.2 s, and *t*_*FNO*_ = 3.35 s, d-f) Higher pressure (large radius) without overshoot due to stable NO dynamics (*t*_*mech*_ + *t*_*NO*_ > *t*_*FNO*_) *p*_*m*_ = 500 Pa, *t*_*Ca*_ = 1 s, *t*_*mech*_ = 0.24 s, *t*_*NO*_ = 0.2 s and *t*_*FNO*_ = 0.21 s. Note the decaying oscillations of the NO concentration in panel d. Near marginal stability (*t*_*mech*_ + *t*_*NO*_ + *t*_*FNO*_) the NO concentrations oscillates at a frequency approximated by *f* = (*E*_1_*K*_*NO*_/*D*)^1/2^/2*π* which corresponds to where the eigenvalues cross the imaginary axis at point B on Fig 4.

Our simulations (Fig 9) show that the dynamic effects of NO are most pronounced when the shear-NO dynamics are unstable. When the shear-NO dynamics are unstable, the radius can overshoot the nominal radius before or after a Ca^2+^-induced contraction, yielding oscillations in radius that are more symmetrical about equilibrium than when shear-NO is stable. At marginal stability (Fig 9d), the NO concentration rings at a frequency determined by the point where the eigenvalues of the shear-NO oscillator cross the imaginary axis *f* = (*E*_1_*K*_*NO*_/*D*)^1/2^/2*π*. At larger radii, the shear-NO mechanism is inherently stable, but can still reduce the magnitude of the oscillations driven by the stretch-Ca^2+^ process. This process is important in the presence of an assisting pressure gradient because the dilation induced by the forced flow can put the vessel into the range of radii where the shear-NO mechanism can inhibit contractions that would otherwise tend to restrict free flow through the vessel.

The overall frequency is set by a complex interplay of stretch-Ca^2+^ and shear-NO mechanisms, but will typically be dominated by the faster of the two processes. Long latency intervals between Ca^2+^-induced contractions can permit NO to produce an unstable dilation, whereas, short intervals due to Ca^2+^ can suppress the autonomous oscillations possible through the NO mechanism. Interestingly, the published clearance rates for Ca^2+^ and NO cover a wide enough range that either possibility exists *in vivo* [15, 32].

**Fig 10:**
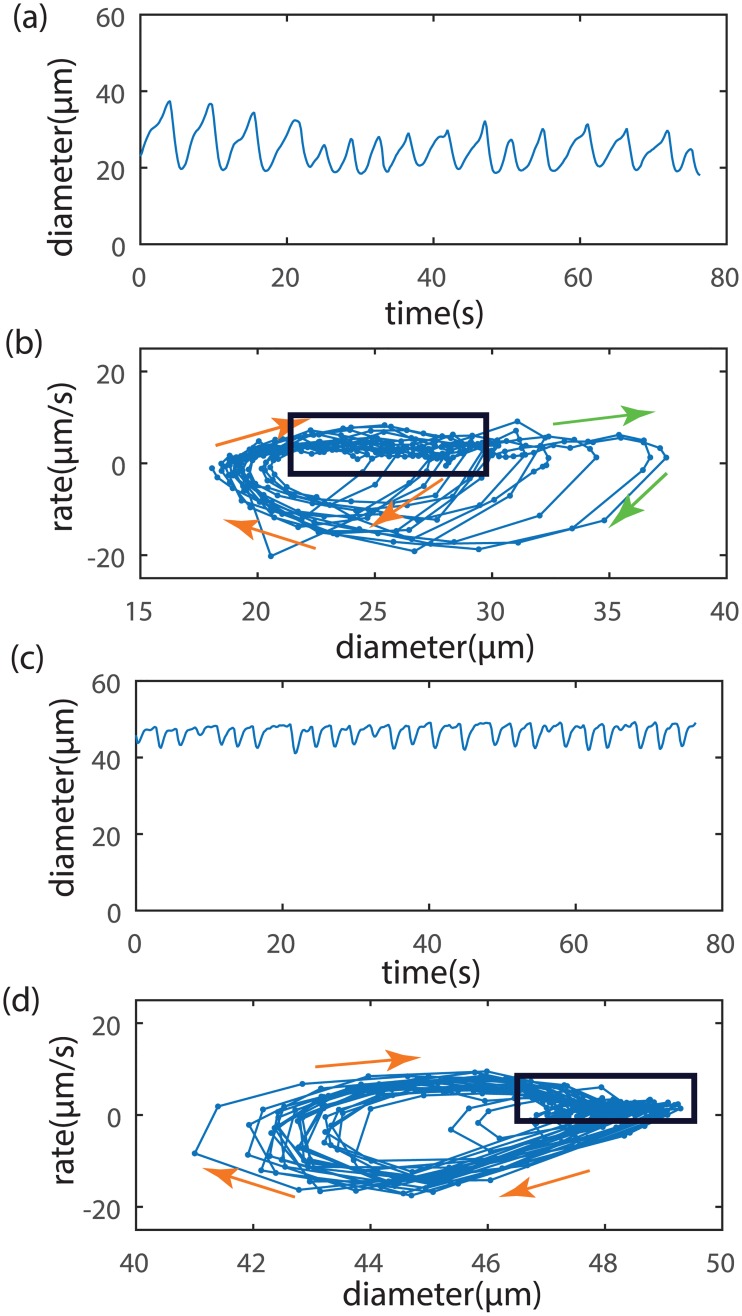
Typical *in vivo* experimental measurements of lymphangion diameter (a,c) in a mouse with phase portraits of diameter vs. rate of diameter change (b,d) [20]. a,b) Wild type mice with Ca^2+^ and NO active. c,d) NO effects genetically deleted in *eNOS*^-/-^ mice. Spacing of data points indicates the rate of motion in the phase plane (sampling period 0.21 s). The box surrounds a region of closely spaced points indicative of an equilibrium condition. Orange arrows show the trajectory during a contraction, while green arrows show a dilation. Contraction and dilation dynamics are generally more erratic when the vessel is small and has active NO than when the vessel is larger and has suppressed NO activity.

Experimental observations of diameter *in vivo* show cycles consistent with the model predictions (Fig 10) (data from [20]). In the absence of direct measurements of concentrations, we employ an alternative phase portrait of diameter plotted against the rate of change of diameter. Fig 10a and 10b are from a wild-type mouse in which Ca^2+^ and NO effects can operate normally. Here, we observe complex oscillations that include both rapid contractions and occasional strong dilations above the baseline diameter as expected from the shear-NO mechanism. In contrast, when the NO effects have been genetically deleted in *eNOS*^-/-^ mice in Fig 10c and 10d, we see wave forms that are nearly identical to each other but dominated by contraction with the dilatory effects of NO appearing to be substantially weakened. In all cases, we see cycles occurring on irregular intervals as we expect from noise-triggered oscillators.

## Discussion

While the correspondence between the experiments and the model is encouraging, we should not expect a model of an isolated lymphangion to reproduce all features of a vessel in an intact, *in vivo* vascular network. For example, the effects of flow introduced from upstream or disturbances from surrounding tissue are inputs present in the animal, but are not included in the modeled dynamics. We have also not yet included effects due to nonlinear valve efficiencies or the bias of the check valves toward the open position [43]. Nonetheless, phase portraits, such as those newly employed here, promise to assist further study of the nonlinear dynamics that govern vascular oscillations.

While our results await further experimental validation and improved estimates of key parameter values, it is interesting to consider the newly identified shear-NO oscillator more generically from a nonlinear dynamics perspective. The shear-NO oscillator has some important similarities and differences from the well-known Van der Pol oscillator [44] which has the form

$$\ddot{x}-A(1-x^{2})\dot{x}+x=g(t)$$ (14)

The form of the characteristic polynomial in Eq 13 implies that the shear-NO oscillator may be written as

$$\ddot{x}+A(1-sgn(\dot{x})B/x^{2})\dot{x}+(x-x_{1})=g(t)$$ (15)

where the generic variable *x* fills the role of the radius in the shear-NO model, *x*_1_ is the nominal operating point and *g*(*t*) is a forcing function that can include steady, random or periodic components.

The stability-determining second term in both oscillators can change sign based on the magnitude of the variable with a positive second term implying stability. The Van der Pol oscillator is known to self-sustain oscillations about the origin in phase space $\left(x,\dot{x})=(0,0\right)$ when *g*(*t*) = 0, as its second term changes sign during different phases of each cycle. In contrast, the shear-NO oscillator operates near $\left(x,\dot{x})=(x_{1},0\right)$, but with *x* > *x*_1_. Therefore, the second term in Eq 15 can be either i) always be positive ($x_{1}^{2}>B$) regardless of the sign on $\dot{x}$ implying inherent stability or ii) can be conditionally positive depending on both the magnitude of B and the sign of $\dot{x}$. As a result, the shear-NO oscillator cannot produce self-sustained oscillations for large radius ($x_{1}^{2}>B$). Furthermore, even when the radius is sufficiently small ($x_{1}^{2}<B$), the radius will return unequivocally to equilibrium as long as $\dot{x}$<0 unless a non-zero forcing function is present to change the sign of $\dot{x}$. However, we find that when $x_{1}^{2}<B$ the magnitude of the forcing needed to start a new cycle can be arbitrarily small and in the form of either random noise or a periodic stimulus provided that enough time has passed for the system to approach its equilibrium point.

In the context of the shear-NO dynamics, the key to oscillations is the inverse dependence on radius for the NO source due to shear stress in Eq 7. As long as the exponent on *R*^−2^ remains negative (increasing radius leading to lower shear stress and less NO production), then the NO-shear mechanism will be capable of a mathematical transition from unstable to stable as seen above in the generic oscillator in Eq 15. The physiological impact of this result then depends on the relative magnitude of the time scales identified herein, not on any single parameter value. For example, the stability of the NO-shear mechanism depends on groups of parameters such as *t*_*FNO*_ = *S*_*NO*_*F*_*NO*_/*E*_1_*K*_*NO*_*R*^2^, which combines the sensitivity of the vessel to shear stress, the contractile force, the wall stiffness and the NO clearance rates.

Inputs of constant magnitude have the effect of adjusting the equilibrium point. Using the shear NO oscillator as an example, an increase in transmural pressure will dilate the vessel, as will a pressure gradient that assists flow by inducing NO production via a steady shear stress. Likewise, a steady source of NO from local inflammation will chronically dilate the vessel [20]. If the vessel becomes sufficiently large, the stability criterion found above suggests that the shear-NO process will not support self-sustaining oscillations, in part due to the direct effect of radius on the stability criterion, but also due to greater stiffness of the wall at larger radius (higher *E*_1_). Nonlinearities in the force production and chemical source/elimination terms may also alter the stability in similar ways.

Numerical simulation and examination in the phase plane reveal that the stretch-Ca^2+^ and shear-NO processes possess numerous symmetries that offer intriguing possibilities when the processes act together (Figs 2 and 6, Table 3). Most notably, we see that the shear-NO process produces rapid and unstable dilation toward a larger radius, followed by stable contraction, while the stretch-Ca^2+^ process causes the vessel to contract rapidly and unstably toward a smaller radius and then to dilate stably. An essential feature of both the Ca^2+^ and NO mechanisms is that taken separately they do not produce traditional, self-sustaining limit cycles, but instead have a one-sided stability near equilibrium from which a new cycle begins only with a perturbation from the local environment. Interestingly, a suitable trigger for the stretch-Ca^2+^ oscillator can be an increase in radius produced by the shear-NO mechanism. And conversely, the shear-NO oscillatory can be triggered by a contraction arising from the stretch-Ca^2+^ mechanism. Balanov et al [45] reviews a variety of similar, so-called “noise-induced” oscillators in contexts outside of lymphatic physiology such as neurons and electrical monovibrators, but to our knowledge, the coupling of symmetric, noise-induced oscillators described in the present study has not been previously investigated.

**Table 3:** Comparison of Ca^2+^-Stretch and NO-Shear Mechanisms Acting Alone.

	Ca^2+^-Stretch	NO-Shear
**Sequence**	Contraction then dilation	Dilation then contraction
**Contraction Speed**	Fast	Slow
**Dilation Speed**	Slow	Fast
**Phase Plane Trajectory**	Clockwise	Counterclockwise
**Contraction Stability**	Unstable	Stable
**Dilation Stability**	Stable	Unstable for small R_1_
**Trigger**	Increasing R	Decreasing R
**Time Constant During Stable Return**	$t_{c}\approx \text{max}(\frac{1}{K_{Ca}},\frac{D}{E_{1}})$	$t_{c}\approx \frac{1}{K_{NO}}+\frac{D}{E_{1}}+\frac{S_{NO}F_{NO}}{E_{1}K_{NO}R_{1}^{2}}$
**Effect of Noise on Period**	Increasing period with smaller noise *T* ≈ *t*_*c*_ log(*C*_*Ca*max_/*σ*)	Increasing period with smaller noise *T* ≈ *t*_*c*_ log(*C*_*NO*max_/*σ*)
**Effect of Radius on Frequency**	Increasing frequency with larger R_1_	Increasing frequency with larger R_1_
**Effect of Radius on Amplitude**	Decreasing amplitude with larger R_1_	Decreasing amplitude with larger R_1_

Balanov et al [45] also review how nonlinear oscillators can synchronize with small-amplitude sinusoidal inputs. Here we found that synchronization of either oscillator can occur over a wide range of frequencies (shear-NO shown in Fig 8, similar behaviors for stretch-Ca^2+^ acting alone and in combination with shear-NO can be observed). The synchronization behavior seen here is similar to that for the forced Van der Pol oscillator in its ability to produce so-called Arnold tongues which are broad domains within which the input and output frequencies are locked in ratios of *m*:*n* where *m* and *n* are small integers [44, 46].

Kornuta *et al* [47] recently showed that lymphatic vessels studied *ex vivo* synchronize their contractions in a 1:1 fashion with imposed oscillatory variations in shear stress when the amplitude of the stimulus is sufficient large and the frequency of the input is relatively close to the autonomous frequency. Interestingly, they also observed that small amplitude variation in transmural pressure did not yield 1:1 frequency locking. However, our examination of their results (Fig 8 in [47]) suggests that 2:3 locking may have occurred. In the absence of imposed flow, they also found that the vessel continued to contract, but with a lower and more erratic frequency consistent with our simulated noise-triggered oscillator (Figs 2 and 3) in the absence of the shear-NO mechanism. Ohhashi *et al* [48] also examined sinusoidal variations in transmural pressure at frequencies well away from the spontaneous frequency. Here too, 1:1 frequency locking did not arise, but the frequency of the contractions responded strongly to the input waveform. Given the subtlety of identifying non-1:1 synchronization, further examination of the experimental record may be warranted.

In conclusion, we have presented a model of a vascular oscillator. The present analysis is sufficiently general to point toward several features that are likely found in other systems. The linear stability analysis shows: (i) complementary mechanisms for dilation and contraction of collecting lymphatic vessels, (ii) a fast, unstable process that recovers slowly and stably to a one-sided equilibrium, (iii) disturbance-based triggering that facilitates either synchronization with a cyclic pacemaker or spontaneous oscillations from random disturbances and (iv) the capability for reciprocal modulation between contractile and relaxation effects. Those features are not only limited to the presented example of Ca^2+^ and EDRFs but can be extended into other fields. The ability of the Ca^2+^ and NO based oscillators to respond to each other and external stimuli explains how lymphatic pumping can be coordinated along extended lengths of collecting lymphatic vessels without the need for higher order coordination. This new class of coupled, noise-driven oscillator can help to explain the diverse pumping behavior of lymphatic vessels.

## References

[1] von der Weid PY, Zawieja DC. Lymphatic smooth muscle: the motor unit of lymph drainage. Int J Biochem Cell Biol. 2004;36(7):1147–53. 10.1016/j.biocel.2003.12.008 15109561

[2] Schmid-Schönbein GW. Microlymphatics and lymph flow. Physiol Rev. 1990;70:987 2217560 10.1152/physrev.1990.70.4.987

[3] Wang W, Nepiyushchikh Z, Zawieja DC, Chakraborty S, Zawieja SD, Gashev AA, et al Inhibition of myosin light chain phosphorylation decreases rat mesenteric lymphatic contractile activity. American journal of physiology Heart and circulatory physiology. 2009;297(2):H726–34. 10.1152/ajpheart.00312.2009 19525378 PMC2724206

[4] Nizamutdinova IT, Maejima D, Nagai T, Bridenbaugh E, Thangaswamy S, Chatterjee V, et al Involvement of Histamine in Endothelium-Dependent Relaxation of Mesenteric Lymphatic Vessels. Microcirculation. 2014;21(7):640–8. 10.1111/micc.12143 24750494 PMC4194136

[5] Zawieja SD, Gasheva O, Zawieja DC, Muthuchamy M. Blunted flow-mediated responses and diminished nitric oxide synthase expression in lymphatic thoracic ducts of a rat model of metabolic syndrome. Am J Physiol-Heart C. 2016;310(3):H385–H93. 10.1152/ajpheart.00664.2015 PMC4796620 26637560

[6] Davis MJ, Donovitz JA, Hood JD. Stretch-activated single-channel and whole cell currents in vascular smooth muscle cells. The American journal of physiology. 1992;262(4 Pt 1):C1083–8. 1373561 10.1152/ajpcell.1992.262.4.C1083

[7] Li CH, Xu QB. Mechanical stress-initiated signal transductions in vascular smooth muscle cells. Cell Signal. 2000;12(7):435–45. 10989277 10.1016/s0898-6568(00)00096-6

[8] Zawieja DC. Contractile physiology of lymphatics. Lymphatic research and biology. 2009;7(2):87–96. 10.1089/lrb.2009.0007 19534632 PMC2925033

[9] Parthimos D, Haddock RE, Hill CE, Griffith TM. Dynamics of a three-variable Nonlinear model of vasomotion: Comparison of theory and experiment. Biophysical journal. 2007;93(5):1534–56. 10.1529/biophysj.107.106278 17483163 PMC1948040

[10] Kapela A, Nagaraja S, Parikh J, Tsoukias NM. Modeling Ca2+ signaling in the microcirculation: intercellular communication and vasoreactivity. Critical reviews in biomedical engineering. 2011;39(5):435–60. 22196162 10.1615/critrevbiomedeng.v39.i5.50 PMC3681513

[11] Kunert C, Baish JW, Liao S, Padera TP, Munn LL. Mechanobiological Oscillators Control Lymph Pumping. Proceedings of the National Academy of Sciences of the United States of America. 2015;112(35):10938–43. 10.1073/pnas.1508330112 26283382 PMC4568261

[12] Cohen RA, Weisbrod RM, Gericke M, Yaghoubi M, Bierl C, Bolotina VM. Mechanism of nitric oxide-induced vasodilatation: refilling of intracellular stores by sarcoplasmic reticulum Ca2+ ATPase and inhibition of store-operated Ca2+ influx. Circulation research. 1999;84(2):210–9. 9933253 10.1161/01.res.84.2.210

[13] Rahbar E, Weimer J, Gibbs H, Yeh AT, Bertram CD, Davis MJ, et al Passive pressure-diameter relationship and structural composition of rat mesenteric lymphangions. Lymphatic research and biology. 2012;10(4):152–63. 10.1089/lrb.2011.0015 23145980 PMC3525898

[14] Meisner JK, Stewart RH, Laine GA, Quick CM. Lymphatic vessels transition to state of summation above a critical contraction frequency. Am J Physiol-Reg I. 2007;293(1):R200–R8. 10.1152/ajpregu.00468.2006 17363681

[15] Shirasawa Y, Benoit JN. Stretch-induced calcium sensitization of rat lymphatic smooth muscle. American journal of physiology Heart and circulatory physiology. 2003;285(6):H2573–7. 10.1152/ajpheart.00002.2003 12946938

[16] Kapela A, Bezerianos A, Tsoukias NM. A mathematical model of Ca2+ dynamics in rat mesenteric smooth muscle cell: agonist and NO stimulation. Journal of theoretical biology. 2008;253(2):238–60. 10.1016/j.jtbi.2008.03.004 18423672

[17] Van Helden DF, Zhao J. Lymphatic vasomotion. Clin Exp Pharmacol Physiol. 2000;27(12):1014–8. 11117222 10.1046/j.1440-1681.2000.03368.x

[18] Haga JH, Li YSJ, Chien S. Molecular basis of the effects of mechanical stretch on vascular smooth muscle cells. Journal of biomechanics. 2007;40(5):947–60. 10.1016/j.jbiomech.2006.04.011 16867303

[19] Hanley CA, Elias RM, Johnston MG. Is Endothelium Necessary for Transmural Pressure-Induced Contractions of Bovine Truncal Lymphatics. Microvascular research. 1992;43(2):134–46. 1584057 10.1016/0026-2862(92)90012-e

[20] Liao S, Cheng G, Conner DA, Huanga Y, Kucherlapati RS, Munn LL, et al Impaired lymphatic contraction associated with immunosuppression. Proceedings of the National Academy of Sciences USA. 2011;108:18784–9. 10.1073/pnas.1116152108 PMC3219138 22065738

[21] Scallan JP, Davis MJ. Genetic removal of basal nitric oxide enhances contractile activity in isolated murine collecting lymphatic vessels. The Journal of physiology. 2013;591(Pt 8):2139–56. 23420659 10.1113/jphysiol.2012.250662 PMC3634525

[22] Rahbar E, Moore JE Jr. A model of a radially expanding and contracting lymphangion. Journal of biomechanics. 2011;44(6):1001–7. 10.1016/j.jbiomech.2011.02.018 21377158 PMC3086717

[23] Rahbar E, Akl T, Cote GL, Moore JE, Zawieja DC. Lymph Transport in Rat Mesenteric Lymphatics Experiencing Edemagenic Stress. Microcirculation. 2014;21(5):359–67. 10.1111/micc.12112 24397756 PMC4174575

[24] Sica D. Calcium channel blocker-related periperal edema: can it be resolved? Journal of clinical hypertension. 2003;5(4):291–4, 7 12939574 10.1111/j.1524-6175.2003.02402.x PMC8099365

[25] Wilson JT, Wang W, Hellerstedt AH, Zawieja DC, Moore JE. Confocal Image-Based Computational Modeling of Nitric Oxide Transport in a Rat Mesenteric Lymphatic Vessel. J Biomech Eng-T Asme. 2013;135(5). 10.1115/1.4023986 PMC3707814 24231961

[26] Frangos JA, Huang TY, Clark CB. Steady shear and step changes in shear stimulate endothelium via independent mechanisms—Superposition of transient and sustained nitric oxide production. Biochemical and biophysical research communications. 1996;224(3):660–5. 10.1006/bbrc.1996.1081 8713104

[27] Kanai AJ, Strauss HC, Truskey GA, Crews AL, Grunfeld S, Malinski T. Shear stress induces ATP-independent transient nitric oxide release from vascular endothelial cells, measured directly with a porphyrinic microsensor. Circulation research. 1995;77(2):284–93. 7614715 10.1161/01.res.77.2.284

[28] Bohlen HG, Gasheva OY, Zawieja DC. Nitric oxide formation by lymphatic bulb and valves is a major regulatory component of lymphatic pumping. Am J Physiol-Heart C. 2011;301(5):H1897–H906. 10.1152/ajpheart.00260.2011 PMC3213974 21890688

[29] Bohlen HG, Wang W, Gashev A, Gasheva O, Zawieja D. Phasic contractions of rat mesenteric lymphatics increase basal and phasic nitric oxide generation in vivo. AJP-Heart and Circulatory Physiology. 2009;297(4):H1319–H28. 10.1152/ajpheart.00039.2009 19666850 PMC2770767

[30] Behnke BJ, Delp MD. Aging blunts the dynamics of vasodilation in isolated skeletal muscle resistance vessels. Journal of applied physiology. 2010;108(1):14–20. 10.1152/japplphysiol.00970.2009 19797684 PMC2885069

[31] Muthuchamy M, Gashev A, Boswell N, Dawson N, Zawieja D. Molecular and functional analyses of the contractile apparatus in lymphatic muscle. Faseb J. 2003;17(3):920-+. 12670880 10.1096/fj.02-0626fje

[32] Thomas DD, Liu X, Kantrow SP, Lancaster JR. The biological lifetime of nitric oxide: implications for the perivascular dynamics of NO and O2. Proceedings of the National Academy of Sciences. 2001;98(1):355. 10.1073/pnas.011379598 PMC14594 11134509

[33] Nagai T, Bridenbaugh EA, Gashev AA. Aging-associated alterations in contractility of rat mesenteric lymphatic vessels. Microcirculation. 2011;18(6):463–73. 10.1111/j.1549-8719.2011.00107.x 21466607 PMC3148320

[34] Bohlen HG, Zhou X, Unthank JL, Miller SJ, Bills R. Transfer of nitric oxide by blood from upstream to downstream resistance vessels causes microvascular dilation. AJP-Heart and Circulatory Physiology. 2009;297(4):H1337–H46. 10.1152/ajpheart.00171.2009 19666847 PMC2770775

[35] Caulk AW, Nepiyushchikh ZV, Shaw R, Dixon JB, Gleason RL. Quantification of the passive and active biaxial mechanical behaviour and microstructural organization of rat thoracic ducts. J R Soc Interface. 2015;12(108). 10.1098/rsif.2015.0280 PMC4528593 26040600

[36] Gashev AA, Zhang RZ, Muthuchamy M, Zawieja DC, Davis MJ. Regional heterogeneity of length-tension relationships in rat lymph vessels. Lymphatic research and biology. 2012;10(1):14–9. 10.1089/lrb.2011.0013 22416912 PMC3357073

[37] Wilkinson DJ. Stochastic Modelling for Systems Biology. 2nd ed Boca Raton, FL: CRC Press; 2012.

[38] Crowe MJ, vonderWeid PY, Brock JA, VanHelden DF. Co-ordination of contractile activity in guinea-pig mesenteric lymphatics. J Physiol-London. 1997;500(1):235–44. 9097947 10.1113/jphysiol.1997.sp022013 PMC1159373

[39] Jamalian S, Davis MJ, Zawieja DC, Moore JE. Network Scale Modeling of Lymph Transport and Its Effective Pumping Parameters. PloS one. 2016;11(2). 10.1371/journal.pone.0148384 PMC4742072 26845031

[40] Strogatz SH. Nonlinear dynamics and Chaos: with applications to physics, biology, chemistry, and engineering. Reading, Mass.: Addison-Wesley Pub.; 1994 xi, 498 p. p.

[41] Kannan MS, Prakash YS, Johnson DE, Sieck GC. Nitric oxide inhibits calcium release from sarcoplasmic reticulum of porcine tracheal smooth muscle cells. The American journal of physiology. 1997;272(1 Pt 1):L1–7. 9038895 10.1152/ajplung.1997.272.1.L1

[42] Cohen RA, Weisbrod RM, Gericke M, Yaghoubi M, Bierl C, Bolotina VM. Mechanism of nitric oxide-induced vasodilatation—Refilling of intracellular stores by sarcoplasmic reticulum Ca2+ ATPase and inhibition of store-operated Ca2+ influx. Circulation research. 1999;84(2):210–9. 9933253 10.1161/01.res.84.2.210

[43] Bertram CD, Macaskill C, Moore JE. Incorporating measured valve properties into a numerical model of a lymphatic vessel. Computer methods in biomechanics and biomedical engineering. 2014;17(14):1519–34. 10.1080/10255842.2012.753066 23387996 PMC4500195

[44] Izhikevich EM. Dynamic Systems in Neuroscience: MIT Press; 2007.

[45] Balanov A, Janson N, Postnov D, Sosnovtseva O. Synchronization: From Simple to Complex. Springer Ser Synerg 2009:1–425.

[46] Kanamaru T. Van der Pol oscillator. Scholarpedia. 2007;2(1).

[47] Kornuta JA, Nepiyushchikh Z, Gasheva OY, Mukherjee A, Zawieja DC, Dixon JB. Effects of dynamic shear and transmural pressure on wall shear stress sensitivity in collecting lymphatic vessels. American journal of physiology Regulatory, integrative and comparative physiology. 2015;309(9):R1122–34. 10.1152/ajpregu.00342.2014 26333787 PMC4666954

[48] Ohhashi T, Azuma T, Sakaguchi M. Active and Passive Mechanical Characteristics of Bovine Mesenteric Lymphatics. American Journal of Physiology. 1980;239(1):H88–H95. 7396023 10.1152/ajpheart.1980.239.1.H88

